# Does Total Gastrectomy Provide Better Outcomes than Distal Subtotal Gastrectomy for Distal Gastric Cancer? A Systematic Review and Meta-Analysis

**DOI:** 10.1371/journal.pone.0165179

**Published:** 2016-10-26

**Authors:** Jin Qi, Peng Zhang, Yanan Wang, Hao Chen, Yumin Li

**Affiliations:** 1 The Second Hospital of Lanzhou University, Lanzhou, Gansu, People's Republic of China; 2 Key Laboratory of Digestive System Tumors of Gansu Province, Lanzhou, Gansu, People's Republic of China; 3 Key Laboratory of Orthopedics of Gansu Province, Lanzhou, Gansu, People's Republic of China; 4 The Evidence-Based Medicine Center of Lanzhou University, Lanzhou, Gansu, People's Republic of China; Xiamen University and Fujian Medical University Affiliated First Hospital, CHINA

## Abstract

**Background/Aims:**

Total gastrectomy (TG) has shown to be superior regarding low risk of recurrence and readmission to distal subtotal gastrectomy (DG) for treatment of distal stomach cancer, but the incidence of postoperative morbidity and mortality in TG cannot be ignored. Therefore, we performed a meta-analysis to compare the effectiveness between TG and DG for distal stomach cancer.

**Methodology:**

A search in PubMed, EMBASE, the Cochrane Library, Web of Science, Chinese Biomedical Database through January 2016 was performed. Eligible studies in comparing of TG and DG for distal gastric cancer were included in this meta-analysis. Review Manager 5.2 software from the Cochrane Collaboration was used for the performance of meta-analysis and STATA 12.0 software for meta-regression analysis.

**Results:**

Ten retrospective cohort studies and one randomized control trial involving 5447 patients were included. The meta-analysis showed no significant difference of postoperative mortality (RR = 1.48, 95%CI = 0. 90–2.44,*p* = 0.12), intraoperative blood loss (MD = 24.34, 95%CI = -3.31–51.99, *p* = 0.08) and length of hospital stay(MD = 0.76, 95%CI:-0.26–1.79, *p* = 0.15). TG procedure could retrieve more lymph nodes than DG(MD = 4.33, 95% CI = 2.34–6.31, *p*<0.0001). According to different postoperative complications, we performed subgroup analysis, subgroup analysis revealed that patients in TG group tended to have a higher rate of postoperative intra-abdominal abscess than DG procedure (RR = 3.41, 95% CI = 1.21–9.63,*p*<0.05). No statistical differences were found in leakage, intestinal obstruction, postoperative bleeding, anastomotic stricture and wound infection between the two groups (*p*>0.05). We pooled the data together, the accumulated 5-year Overall Survival rates of TG and DG groups were 49.6% (919/1852) vs.55.9%(721/1290) respectively. Meta-analysis revealed a favoring trend to DG procedure and there was a statistical difference between the two groups (RR = 0.91,95% CI = 0.85–0.97,*p* = 0.006).

**Conclusion:**

Based on current retrospective evidences, we found that in spite of similar postoperative mortality, TG for distal gastric cancer provided a high risk of five-year Overall Survival rate. DG procedure can be a recommendation for distal gastric cancer, whereas due to lack of high quality RCTs in multicenter and the relatively small sample size of long-term outcomes, further comparative studies are still needed.

## Introduction

Gastric cancer is the second leading cause of cancer deaths worldwide with an estimated incidence of 870000 per year nearly two-thirds of cases occurring in the developing countries[1]. Surgical resection is the only therapy and an option to enhance the survival rate of patients with gastric cancer[2]. The extent of gastrectomy for curative treatment of gastric cancer depends on tumor location, tumor size and tumor stage[3,4]. However, the distal subtotal gastrectomy and total gastrectomy for centuries, there has been controversy about the choice of the best surgical procedure for the distal half of gastric cancer which is usually resection by the distal subtotal gastrectomy in china[5]. Although total gastrectomy can maximumly reduce gastric remnant cancer[6], it leads to the postoperative limited diet, dysphagia, dry mouth, and reflux symptoms which will affect the patient's quality of life [7]. Whether distal subtotal gastrectomy and total gastrectomy is the same in perioperative period, complications and long-term survival rate or not, different studies have different results. The purpose of this meta-analysis is to evaluate which surgical procedure is the superior surgical treatment for the distal half of gastric cancer, concerning operation time, intraoperative blood loss, hospital stay, postoperative mortality and five-years overall survival rate, as well as the patient’s quality of life, etc.

## Methods

### Search strategy

Trials were identified by searching PubMed, EMBASE, the Cochrane Library, Web of Science, Chinese Biomedical Database through January, 2016, Search strings of PubMed were (“gastric cancer” (Mesh) AND “carcinoma” (Mesh)) AND “total gastrectomy” (Mesh) AND (“distal gastectomy”(Text word) OR “distal subtotal gastectomy”(Text word) OR “distal resection” (Text word) OR “partial gastrectomy” (Text word)). Relative reference lists were also screened for relative articles. All the searches were conducted independently by two investigators(JQ and YNW). Discrepancies in the interpretation were resolved by discussion.

### Inclusion and exclusion criteria

We only identified studies comparing Total versus Distal Gastrectomy for Gastric Cancer. Either prospective or retrospective controlled studies were eligible. For all gastric cancer patients who had undergone gastrectomy or laparoscopic-assisted gastrectomy were either distal subtotal gastrectomy(DG) or total gastrectomy(TG). The primary outcome measure were mortality and five-year Overall Survival(OS), while secondary outcomes were operation time, intraoperative blood loss, harvested lymph nodes, hospital stay, quality of life, postoperative complication including wound infection, leakage, anastomotic stenosis, intestinal obstruction, intra-abdominal abscess, etc.

We excluded studies which did not report the baseline information between DG and TG groups. Article of too small size, failure to meet the inclusion criteria and data unusable were excluded. Of course, duplicated studies were identified for exclusion.

### Date extraction and Quality Assessment

The data was extracted and critically appraised independently by two authors. We extracted operative time, intraoperative blood loss, hospital stay, postoperative mortality, five-year overall survival were used to compare the postoperative recovery of the procedures. The postoperative complications including wound infection, anastomotic leakage, anastomotic stricture, intestinal obstruction, intra-abdominal abscess and bleeding were compared. The hospital mortality, five-year overall survival rate were used to estimate the postoperative safety of DG versus TG.

Newcastle–Ottawa Quality Assessment Scale for cohort studies was used for assessing the quality of non-randomized studies included in this meta-analyses(Table 1)[8]. Using the tool, each study is judged on eight items, which were used to assess patient population and selection, study comparability, follow-up, and the outcome of interest. A star system is used to allow a semi-quantitative assessment of studies which are awarded a maximum of one star for each item in the assignment of two stars. The NOS stars are added up to compare the study quality. Each study was graded as either low quality (0–5) or high quality (6–9). The methodological quality of included studies is shown in Table 2, the most of low-quality studies were excluded.

**Table 1:** Newcastle-Ottawa quality assessment scale*.

Selection
1) Representativeness of the exposed cohort
a) truly representative of the average __GC Patient__ in the community
b) somewhat representative of the average __GC Patient__ in the community
c) selected group of users eg nurses, volunteers
d) no description of the derivation of the cohort
2) Selection of the non exposed cohort
a) drawn from the same community as the exposed cohort
b) drawn from a different source
c) no description of the derivation of the non exposed cohort
3) Ascertainment of exposure
a) secure record (eg surgical records)
b) structured interview
c) written self report
d) no description
4) Demonstration that outcome of interest was not present at start of study
a) yes ¯**¯**
b) no
Comparability
1) Comparability of cohorts on the basis of the design or analysis
a) study controls for _age, sex, BMI_ **¯**
b) study controls for any additional factor (tumor size, stage, etc.)
Outcome
1) Assessment of outcome
a) independent blind assessment ¯**¯**
b) record linkage ¯**¯**
c) self report
d) no description
2) Was follow-up long enough for outcomes to occur
a) yes (5 years) **¯**
b) no
3) Adequacy of follow up of cohorts
a) complete follow up—all subjects accounted for ¯ **¯**
b) subjects lost to follow up unlikely to introduce bias—small number lost—> _90_ % (select an adequate %) follow up, or description provided of those lost) ¯**¯**
c) follow up rate < _60_% (select an adequate %) and no description of those lost
d) no statement

* A study can be awarded a maximum of one star for each numbered item within the Selection and Outcome categories. A maximum of two stars can be given for Comparability

GC, gastric cancer; BMI, body mass index.

**Table 2:** Quality assessment of included studies.

References	selection				comparability	outcome			score
	1	2	3	4	5	6	7	8	
Bozzetti F[9]	*	*	*	*	**	*		*	8
Bozzetti F[10]	*	*	*	*	**	*	*	*	9
Gockel I[11]	*	*		*	**		*	*	7
Moghimi M[12]	*	*		*	**			*	6
Lee SE[13]	*	*		*	*	*		*	6
Jang YJ[14]	*	*	*	*	*	*	*		7
Mocan L[15]	*	*	*	*	*	*	*		7
Park SJ[16]	*	*	*	*	**	*			6
Kim DJ[7]	*	*	*	*	*				5
Lin JX[6]	*	*		*	**	*		*	7
Liu Z[17]	*	*		*	**			*	6

### Statistical analysis

Data was analyzed using Review Manager 5.2 software programs(Cochrane Collaboration) and STATA 12.0. For dichotomous scales, the data from these comparative trials were expressed as risk ratio (RR) along with 95% confidence interval (CI). If there were continuous data of measurement, the mean difference (MD) was used as the measure of association. Effects on quantitative measures(e.g. operation time, blood loss) were evaluated by mean difference (MD) approach. Five-year OS were analyzed by pooled hazard ratios (HR) and their 95% confidence intervals (CI). HRs and their95% CIs for five-year OS rate was obtained by used the published methods to calculate them. Inverse Variance (IV) test was used for MD estimate. Date was pooled using the fixed-effect model but the random-effects model was also considered to ensure robustness of the model. The heterogeneity among studies was performed using the I-squared index(*I*^*2*^) statistic[18]. When the heterogeneity was high (*I*^*2*^>50%),we used random-effects model to analysis. Otherwise a fixed-effect model was used. All the *p* values were two-tailed with significance level of 0.05,except for the heterogeneity test (*p* = 0.10).

## Results

### Literature search and selection

**Fig 1:**
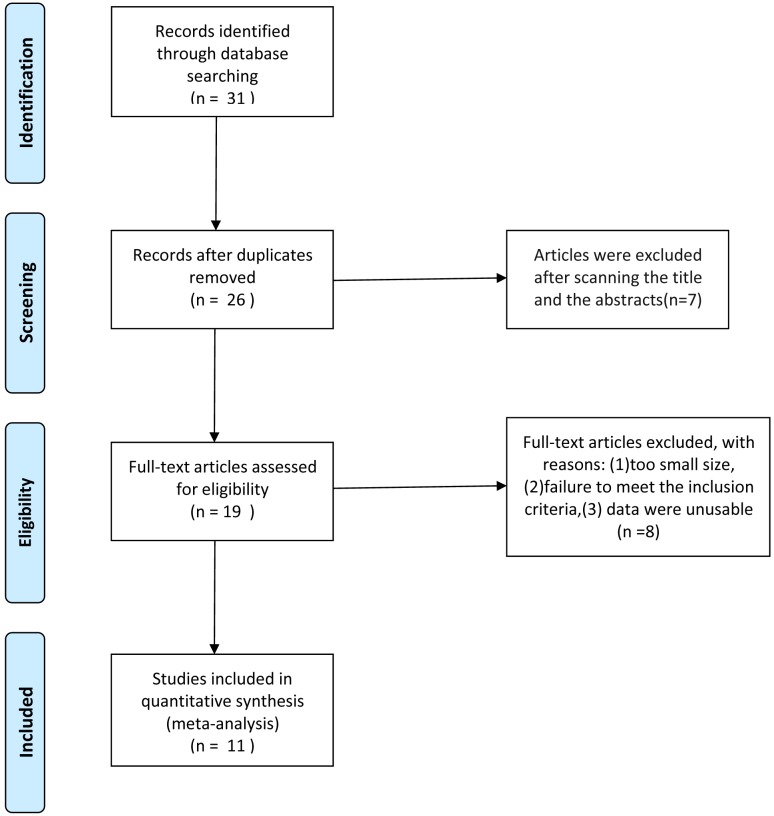
PRISMA flow diagram of study selection.

The initial search revealed 31 citations, and 19 potentially eligible articles were secondarily selected by reading the full-text and 8 articles were excluded because of a small size, failure to meet the inclusion criteria, data were unusable. The flow diagram of reviews shows the detailed process of selection (Fig 1). Finally, 11 studies [6,7,9–17] involving 5447 patients (2418 by TG vs 3029 by DG) were included for our analysis which included Laparoscopy-assisted and open gastrectomy for gastric cancer (Table 3). The baseline characteristics between both groups were shown in Table 4.

**Table 3:** Details of the articles included in the meta-analysis.

Studies	Year	Country	Journal	Sample size	Type of study
				TG/DG	
Bozzetti F[9]	1997	Italy	Ann. Surg	304/320	Randomized control trail
Bozzetti F[10]	1999	Italy	Ann. Surg	303/315	Randomized control trail
Gockel I[11]	2005	Germany	Langenbecks Arch Surg	240/80	Retrospective cohort study
Moghimi M[12]	2008	Iran	Chin J cancer Res	35/31	Retrospective cohort study
Lee SE[13]	2009	Korea	J SurgOncol	67/473	Retrospective cohort study
Jang YJ[14]	2010	Korea	J Surg Oncol244/158	178/148	Retrospective cohort study
Mocan L[15]	2013	Romania	J Gastrointestinliver Dis	89/91	Retrospective cohort study
Park SJ[16]	2014	Korea	J Gastric cancer	61/214	Retrospective cohort study
Kim DJ[7]	2015	Korea	Surg Endosc	94/569	Retrospective cohort study
Lin JX[6]	2015	China	Surg Endosc	976/646	Retrospective cohort study
Liu Z[17]	2015	China	Chin J Gastrointest Surg	71/142	Retrospective cohort study

**Table 4:** Characteristics of the articles included in the meta-analysis.

Studies	Approach	Age(years)	Male/Female	BMI(Kg/m2)	Tumor diameters(cm)	Tumor depth				Number of metastatic LN				Stage(No.)				Extension of surgery				Lymphadenectomy		Follow-up (Months)
						T1	T2	T3	T4a	N0	N1	N2	N3	I	II	III	IV	None	Spleen	Other organs or multiple	Spleen and orther organs	D1	D2	
BozzettiF[9]	TG		175/129							130								221	56	11	16	0	304	
	DG		187/133							157								288	17	12	3	0	320	
BozzettiF[10]	TG		174/129			74	73	149	7	126	112	44	19	104	68	75	54	220	56	11	16	0	303	75
	DG		183/132			96	74	138	7	155	96	45	19	134	65	58	58	286	15	11	3	0	315	72
Gockel I[11]	TG		156/84			36	109	70	25	67	58	90	25						153	9		0	240	120
	DG		54/26			22	34	17	7	29	21	25	5						1	0		0	80	
MoghimiM[12]	TG	58.75±7.2	20/15																			0	35	20
	DG	63.06±5.1	21/10																			0	31	
Lee SE[13]	LTG	52.3±13.6	39/28	22.9±2.9	4.0±2.9	51	16	0	0	56				61	6							32	35	
	LDG	57.0±12.1	266/207	24.0±3.0	3.1±1.8	428	45	0	0	414				453	20							199	274	
Jang YJ[14]	TG	53.42±13.07	101/77		6.52±3.39									17	37	89	35					44	134	60
	DG	54.41±13.65	95/53		4.04±2.11									36	41	62	9					49	99	
Mocan L[15]	TG	63.5±8.1	58/31			24	24	29	12	44	25	15	4	25	25	48		9	15	24	29	25	64	
	DG	63.5±8.1	49/42			30	16	38	7	38	29	17	7	35	18	38		8	22	38	16	42	49	
Park SJ[16]	TG	56.9±12.1	41/20	23.8±2.9										55	5	1							61	24
	DG	59.2±11.1	121/93	23.8±2.8										202	12	0							214	
Kim DJ[7]	LTG	61.4±12.3	67/27		5.0±3.3	37	14	21	22	61	6	10	17	46	22	26						17	77	
	LDG	61.9±12.0	348/221		3.0±2.2	409	60	63	37	446	46	39	38	432	75	62						232	337	
Lin JX[6]	LTG	62.4±10.8	766/210	21.9±2.9	5.9±2.6	128	357	491	0	214	158	175	429	72	252	652								32
	LDG	60.2±10.7	444/202	22.1±3.2	4.5±1.8	136	223	287	0	144	88	158	256	69	165	412								
Liu Z[17]	TG	54.3±11.0	51/20			9	9	26	28	19	11	15	26	10	19	42								20
	DG	55.8±11.9	101/41			16	18	52	56	41	44	25	32	22	52	68								

### Results of the meta-analysis

#### Operative findings

**Fig 2:**
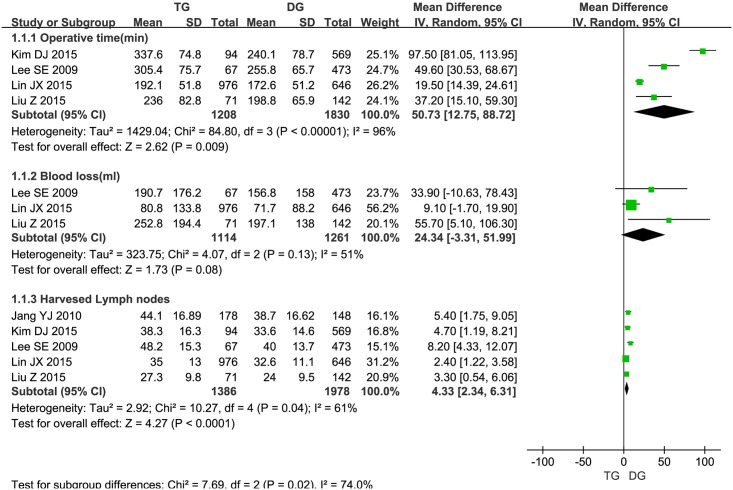
Meta-analysis of surgical outcomes between TG and DG for gastric cancer.

Three articles [6,13,17] reported blood loss and four studies [6,7,13,17]reported operation time. Meta-analyses showed that DG took obviously shorter operative time than TG procedure (MD = 50.73, 95% CI: 12.75–88.72, *p* = 0.009), heterogeneity was observed (*p*<0.00001; *I*^*2*^ = 96%), so we used a random-effect model for this analysis. DG involving 1261 patients took less volume of intraoperative blood loss than TG procedure involving 1114 patients, but no statistical differences were found between the two groups, (MD = 24.34, 95% CI = -3.31–51.99, *p* = 0.08) (Fig 2). There was significant heterogeneity among studies (*p* = 0.13; *I*^*2*^ = 51%), so we used a random-effect model here(Fig 2). Five studies [6,7,13,14,17] reported harvested lymph nodes, meta-analysis confirmed that TG procedure could retrieve more lymph nodes than DG (MD = 4.33, 95% CI = 2.34–6.31, *p*<0.0001) (Fig 2).

#### Length of hospital stay

**Fig 3:**
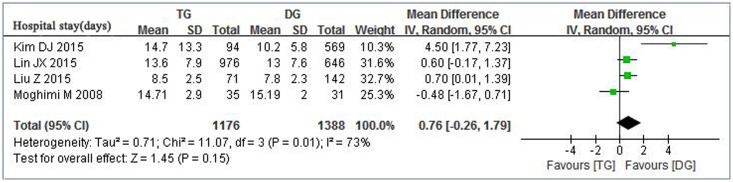
Meta-analysis of Length of hospital stay between TG and DG for gastric cancer.

There was significant heterogeneity (*p* = 0.01; *I*^*2*^ = 73%) between studies, so we used a random-effect model for the meta-analysis of length of hospital stay. Data regarding hospital stay were provided in four studies [6,7,12,17] involving 2559 patients, meta-analysis showed that there was no significant difference in the length of hospital stay between TG and DG group (MD = 0.76, 95% CI:-0.26–1.79, *p* = 0.15) (Fig 3).

#### Postoperative complication

**Fig 4:**
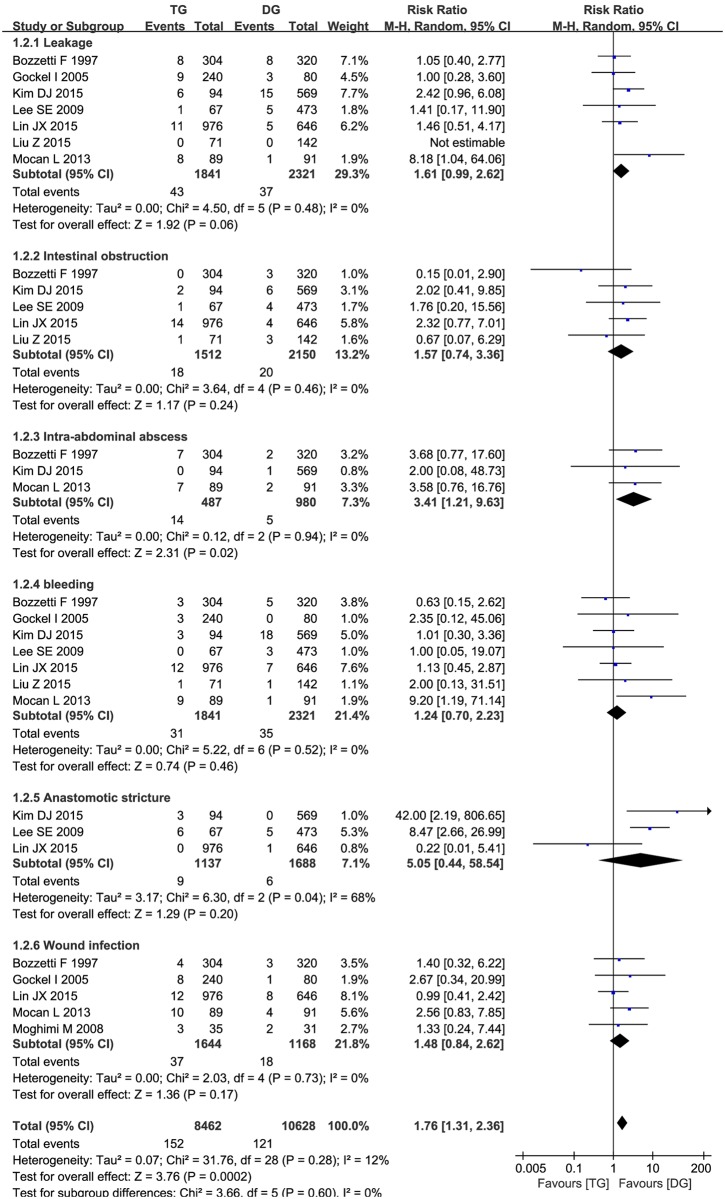
Meta-analysis of postoperative complication between TG and DG for gastric cancer.

Meta-analysis on 8 observational studies [6,7,9,11–13,15,17] showed that patients after TG group experienced significantly higher total postoperative complication risk compared to DG procedure. (RR = 1.76, 95% CI = 1.31–2. 36,*p* = 0.0002) (Fig 4). According to different postoperative complications, we performed subgroup analysis, subgroup analysis revealed that patients in TG group tended at a higher rate of postoperative intra-abdominal abscess than DG procedure (RR = 3.41, 95% CI = 1.21–9.63,*p*<0.05) (Fig 4). No statistical differences were found in leakage, intestinal obstruction, postoperative bleeding, anastomotic stricture and wound infection between the two groups (*p*>0.05) (Fig 4). It implied a trend of potential survival benefit of TG procedure for distal gastric cancer.

#### Postoperative mortality and Overall survival

**Fig 5:**
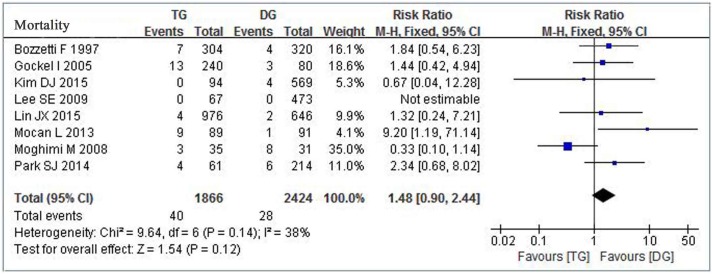
Meta-analysis of postoperative mortality between TG and DG for gastric cancer.

Date regarding to patient 30-day mortality at postoperation were reported in eight studies[6,7,9,11–13,15,16] involving 1866 patients of TG and 2424 patients of DG. The accumulated mortality rates of TG and DG groups were 2.14% (40/1866) and 1.16% (28/2424) respectively. Although TG took higher accumulated mortality rate than DG group, meta-analysis revealed there was no significant difference on postoperative mortality. (RR = 1.48, 95% CI = 0. 90–2.44,*p* = 0.12) (Fig 5).

**Fig 6:**
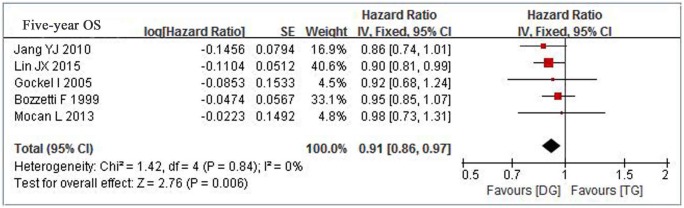
Meta-analysis of five-year overall survival rate between TG and DG for gastric cancer.

The long-term Overall Survival (OS) is an important outcome to assess the safety of the operation type. Five articles[6,10,11,14,15] reported the five-year OS of both procedures. We pooled the data together, the accumulated 5-year OS rates of TG and DG groups were 49.6% (919/1852) vs.55.9%(721/1290)respectively. Meta-analysis revealed a favoring trend to DG procedure, and there was statistically difference between the two groups (HR = 0.91,95% CI = 0.85–0.97,*p* = 0.006) (Fig 6).

#### Meta-regression

We examined the outcome variables with high heterogeneity (I^2^ >50%) in a meta-regression model. The analyses indicated that study quality, sample size, year of publication, country of patients, and stage of gastric cancer were significant sources of heterogeneity (Table 5).

**Table 5:** Meta-regression analysis.

Variable	Coef.	Std. Err.	p value	95% Conf.Interval
**Operative time**				
Study quality	-37.321	7.013	0.034	-67.494 to -7.146
Simple sizes	0.022	0.107	0.857	-0.4391 to 0.483
year of publication	0.257	7.981	0.977	-34.085 to 34.598
country of patients	-46.490	25.357	0.208	-155.595 to 62.614
stage of gastric cancer	42.472	35.851	0.358	-111.782 to 196.725
**Blood loss**				
Study quality	-34.315	17.923	0.306	-262.054 to 193.423
Simple sizes	-0.029	0.014	0.297	-0.213 to 0.155
year of publication	-1.387	6.972	0.875	-89.974 to 87.200
country of patients	-8.324	41.831	0.875	-539.844 to 523.196
stage of gastric cancer	34.315	17.923	0.306	-193.423 to 262.054
**Harvesed Lymph nodes**				
Study quality	-0.879	1.445	0.586	-5.479 to 3.720
Simple sizes	-0.002	0.002	0.337	-0.007 to 0.003
year of publication	-0.749	0.265	0.066	-1.591 to 0.093
country of patients	-3.445	1.214	0.066	-7.308 to 0.418
stage of gastric cancer	1.73	2.035	0.458	-4.747 to 8.206
**Hospital stay**				
Study quality	-0.912	1.996	0.679	-9.111 to 7.287
Simple sizes	0.001	0.002	0.675	0.008 to 0.010
year of publication	0.458	0.115	0.058	-0.037 to 0.953
country of patients	1.233	2.523	0.674	-9.648 to 12.115
stage of gastric cancer	-0.216	3.054	0.950	-13.354 to 12.922
**Anastomotic stricture**				
Study quality	-2.582	1.112	0.259	-16.712 to 11.548
Simple sizes	0.012	0.005	0.245	-0.048 to 0.071
year of publication	-0.176	0.724	0.848	-9.377 to 9.025
country of patients	-3.957	1.732	0.263	-25.962 to 18.047
stage of gastric cancer	3.115	3.337	0.522	-22.746 to 45.519

#### Publication Bias

Funnel plots and Egger’s weighted regression test were used to assess the publication bias. When the number of included studies was less ten, we did not assess the publication bias, otherwise, it could have a big bias[19]. In our study, we did not performed the publication bias, because funnel plots test was advisable in the event of at least ten individual studies.

## Discussion

This is the first meta-analysis focusing on the surgical outcomes, postoperative morbidity and long-term effects of TG and DG surgical treatment in gastric cancer patients. From a surgical point of view, the best choice for surgical procedure in distal stomach cancer is still controversial. USA surgeons usually perform TG for cancer of the distal stomach[20]. Studies have shown that TG did not increase postoperative hospital stay, mortality and even morbidity in comparison with DG. Moreover, DG procedure have higher risk of recurrence and readmission than that of TG, consequently a great number of second surgeries in these gastric cancer patients[21]. While in most European countries, DG was the general procedure of choice[22,23]. They regarded that the incidence of postoperative morbidity and mortality in TG was at least two times higher than that of DG procedure[13,24,25]. Studies have reported similar short- and long-term outcome between these two surgical procedures[10,26,27]. According to “Japanese gastric cancer treatment guideline 2010”, the standard surgical procedure for clinically node-positive or T2-T4a tumors is either total or distal gastrectomy. DG is selected when a satisfactory proximal resection margin can be obtained[28]. But it is difficult to assess whether the tumor cell remain or not in the proximal resection margin, we therefore compared operation time, intraoperative blood loss, retrieved lymph nodes, postoperative morbidity, 30-day mortality and five-year OS rate after TG or DG surgical procedure in patients with gastric cancer. Because the quality of our included studies was scaled by NOS and most of the clinical characteristics were matched, the two groups(TG vs DG) were comparable. Based on our study, we found that operative time of TG procedure was longer than DG group and the difference has statistically significance. However, blood loss during the operation tended to have an increase in TG group, but TG procedure could retrieve more lymph nodes than DG. To some extent, although the complexity and trauma of TG was the main reason, the number of harvested lymph nodes in TG group, which was more than that in DG group(MD = 4.33, 95% CI = 2.34–6.31, *p*<0.0001) was regarded as surgically acceptable. We supposed that with the technological improvement and the development of the instruments, the volume of intraoperative blood loss of TG procedure seemed to be more than that of DG, but the reduction of the operative time and blood loss has been observed in TG procedure. The postoperative complication was an important outcome to assess the safety of the operation type. In the subgroup-analysis, we found the total postoperative complications tended to be less in DG group which was associated with relatively minor trauma. However, it was difficult to ensure that the proximal resection margin was without gastric cancer cell residue by DG. Interestingly, in our meta-analysis revealed that the accumulated five-year OS rate was lower in TG group (49.6% vs. 55.9%). And there was statistical difference indeed and the five-year OS results favored the DG group. It implied a trend of potential survival benefit of TG procedure for distal gastric cancer.

With the improvement of laparoscopic techniques and the development of laparoscopic instruments, laparoscopic gastrectomy has been widely performed in the world for its benefits over open surgery such as less blood loss, less postoperative pain, quicker bowel function recovery, shorter hospital stay and lower postoperative morbidity except longer operative time[29,30]. So in this meta-analysis, we included studies with compared Laparoscopic-assisted DG and TG for analysis. The short or long term outcomes of laparoscopic assisted gastrectomy consisted that of open procedure. With the improvement of surgical techniques and the development of the instruments, the number of long-term survivors after resection for gastric cancer has been increasing and their QoL has become an important issue. In this study, due to lacking of QoL questionnaire standard scales, several articles we searched in comparison to short- or long-term QoL after undergone TG and DG could not be system evaluated[16,27,31–36]. Studies have shown that patients who undergone DG have a better QoL than those who undergone TG in shroter postoperative follow-up period[36]. However, along with time frame, these differences diminished whether patients underwent TG or DG[16,32,35]. Jentschura D et al. reported that there were no differences between aged and younger patients indicates that age alone is no contraindication to major surgery, among the long-term survivors aged patients can have the same postoperative QoL as young people[31]. As we all know, the digestive function has important effect on the clinical outcomes and quality of life. EijiNomura investigated digestive functions of gastric cancer patients and found that postoperative functional outcomes were not affected by the manner of reconstruction, but by the size of the remnant stomach[37]. It was inevitable that TG procedure would yield worse complications such as oesophageal reflux, diarrhea, and nausea/vomiting because of a restricted food reservoir in the TG group. Lee SS regarded that survivors after TG exhibited ongoing QoL inferiority on various functional and symptom scales at postoperative five years, beyond that time, QoL inferiority of the TG to the DG group generally disappeared except of eating restrictions implicates[34]. It is possible that some form of gastric substitute, such as jejunal interposition, might also be helpful in reducing eating restrictions following TG in the longer term[38].

Although this meta-analysis study was strictly executed according to the quality of reporting meta-analysis statement[39],there are several limitations to our meta-analysis. Firstly, the methodological quality of studies was not optimal, just only two RCTs were included in our study. Secondly, the relative small sample size in short- and long-term outcomes made our conclusion not convincible enough, more studies focusing on this subject are still needed. Lastly, The studies included were just conducted in Italy, Germany, Iran, Korea, Romania and China, whereas many centers in the rest of the world have not been included in this study. Therefore, we still need more high-quality, multicenter, randomized, controlled trials from other countries and regions.

In conclusion, In spite of similar 30-day mortality and long-term QoL compared with TG procedure, DG procedure for distal gastric cancer have advantages of less operative time, less blood loss, quicker postoperative recovery, relative higher OS rate, which is feasible and recommended surgery for distal gastric cancer in locally early and advanced stages. However, high quality RCTs in multicenter and the comparative studies are still needed for further validation.

## Supporting Information

S1 PRISMA Checklist PRISMA checklist. (DOC)
